# Early‐Stage Glottic Squamous Cell Carcinoma: A Nationwide Analysis on Incidence, Survival, Recurrences, and Laryngectomies After Radiotherapy in the Netherlands (2015–2021)

**DOI:** 10.1002/hed.70245

**Published:** 2026-03-25

**Authors:** Sabine M. L. Linden, Boukje A. C. van Dijk, Marielle E. P. Philippens, Gerben E. Breimer, Remco de Bree, Johannes A. Rijken, Mischa de Ridder

**Affiliations:** ^1^ Department of Radiotherapy University Medical Center Utrecht Utrecht the Netherlands; ^2^ Department of Epidemiology University Medical Center Groningen Groningen the Netherlands; ^3^ Department of Research and Development Netherlands Comprehensive Cancer Organisation (IKNL) Utrecht the Netherlands; ^4^ Department of Pathology University Medical Center Utrecht Utrecht the Netherlands; ^5^ Department of Head and Neck Surgical Oncology University Medical Center Utrecht Utrecht the Netherlands

**Keywords:** early‐stage glottic cancer, head and neck cancer, laryngectomies, oncological outcome, radiotherapy

## Abstract

**Objective:**

Growing interest in new radiotherapy strategies for early‐stage glottic cancer highlights the importance of reviewing current treatment outcomes. This nationwide study presents the incidence, survival, recurrences, and laryngectomies for these patients.

**Methods:**

Patients diagnosed with cT1‐2N0M0 glottic squamous cell carcinoma (SCC) between 2015 and 2021 in the Netherlands were included. Patients diagnosed between 2015 and 2019 were analyzed for survival, recurrences, and laryngectomies after radiotherapy treatment.

**Results:**

A total of 2214 patients were diagnosed with early‐stage glottic SCC. A total of 826 patients were treated with radiotherapy between 2015 and 2019. The 5‐year overall survival, relative survival, and recurrence‐free survival after radiotherapy were 75%, 88%, and 86%, respectively. Of the 826 patients, 47 (6%) underwent laryngectomy. Only four (0.5%) patients had a laryngectomy due to severe radiation toxicity.

**Conclusion:**

This nationwide study demonstrates that radiotherapy for early‐stage glottic SCC results in excellent oncological outcomes. Radiation‐induced laryngectomies for this treatment are extremely rare.

## 1 Introduction

Glottic squamous cell carcinoma (SCC) often presents at an early stage (cT1a, cT1b, or cT2), as even small irregularities on the vocal cord can cause hoarseness. This hoarseness is an early clinical symptom of the disease. In more advanced cases, symptoms may include sore throat, painful swallowing, and dysphagia. Risk factors for laryngeal cancer include tobacco smoking and alcohol consumption [1, 2, 3], and the disease is significantly more prevalent in males than in females [4]. Diagnosis of glottic SCC typically involves physical examination using flexible endoscopy to visualize the lesion.

Current treatment strategies for early‐stage glottic SCC are transoral microscopic laser surgery or radiotherapy. These treatment strategies result in similar oncological outcomes for cT1a tumors [5]. The treatment choice depends on patient and tumor‐related factors [6] and hospital resources. In Dutch clinical practice, smaller tumors (cT1a) are typically treated with microscopic laser surgery, whereas more extensive tumors (cT1b and higher) are treated with primary radiotherapy. In primary radiotherapy, we often choose between two fractionation schedules: A mildly hypofractionated schedule (25 fractions of 2.4 Gy) or a conventional fractionation schedule (30–35 fractions of 2 Gy). The conventional schedule is more often used for larger T2 tumors since these patients also receive elective irradiation of the neck lymph nodes. The mildly hypofractionated schedule is used to treat cT1 and small cT2 tumors, targeting the tumor alone.

An update on the outcomes of radiotherapy treatments in the Netherlands is needed to evaluate the feasibility and safety of new radiotherapy treatment strategies, such as extreme hypofractionation. This large nationwide cohort represents all institutes within the Netherlands. The data is linked to the pathology database for an evaluation of severe toxicity outcomes. This study provides the incidence, survival, recurrences, and laryngectomies for patients with early‐stage glottic cancer who are treated in the Netherlands.

## 2 Methods

### 2.1 Data Collection

This study utilized population‐based data from the nationwide Netherlands Cancer Registry (NCR) for the period 2015–2021. Inclusion criteria were as follows: (1) patients diagnosed with cT1‐2N0M0 glottic SCC (ICD‐O‐3 C32.0), (2) age of 18 years or older, and (3) no prior malignancies in the head and neck region. Each included patient and tumor was assigned to a unique identification number. The dataset included variables such as year of diagnosis, clinical tumor stage, age at diagnosis, sex, treatment details, smoking status and alcohol consumption at time of diagnosis, details on first recurrence, and vital status. Follow‐up on vital status was recorded from the date of diagnosis until death, emigration, or until February 1, 2024, which corresponds to the date of linkage with the Dutch Population Registry (Basisregistratie Personen, BRP).

For incidence analysis, the entire population was used, including patients who received radiotherapy, surgery, or no treatment. Patients treated with primary radiotherapy and diagnosed between 2015 and 2019 were selected for further analyses to ensure a 5‐year follow‐up. To assess the number of patients who needed a laryngectomy, supplementary pathology data were retrieved from the nationwide network and registry of histo‐ and cytopathology in the Netherlands (PALGA) [7]. This database provides pathology reports and was linked to the NCR data based on gender, age, and date.

### 2.2 Study Variables

The variables selected for analysis included age, sex, clinical tumor stage, smoking status, alcohol consumption, and fractionation schedule. Based on total dose and number of fractions, the fractionation schedules were classified into two groups: 2 Gy/fx (conventional radiotherapy) and > 2 Gy/fx (hypofractionated radiotherapy). Alcohol abuse was defined as > 3 units per day. Pathology reports were manually searched for laryngectomies. Resected larynxes in which no recurrence was reported in the pathology records were classified as laryngectomies due to radiotherapy toxicity.

### 2.3 Statistical Analysis

To evaluate the trend in incidence rates over time, the revised European standardized rates (RESR) per 100 000 persons per year were calculated using the revised European standard population [8], enabling comparisons over time while accounting for the Dutch population size and age composition. Joinpoint trend analysis software version 5.2.0 [9] was used to analyze trends in the standardized incidence rates. These trends were reported as an annual percentage change (APC) with 95% confidence intervals (95% CIs).

All the other analyses were performed using Stata/SE version 17.0. The total population and the population treated with radiotherapy were included in the descriptive analysis on patient characteristics. Numbers and proportions are reported for included variables. The median and 25th and 75th percentiles (p25–p75) were used for non‐normally distributed data. For patients receiving radiotherapy, the Kaplan–Meier method was used to estimate survival rates. The relative survival was defined as the ratio of the observed survival rate in the study population to the survival rate in the Dutch population (accounting for age, sex, and year) [10, 11]. The relative excess risk (RER) was estimated using univariable and multivariable generalized linear models. Recurrence‐free survival (RFS) was defined as survival without local, regional, or metastatic recurrence during the 5‐year follow‐up period. A Cox proportional hazards model was used to estimate the hazard ratio (HR) for univariable and multivariable analyses. Variables were included in the multivariable analysis if they had a *p* < 0.10 in the univariable analysis. A *p* < 0.05 was considered statistically significant for all tests.

## 3 Results

**FIGURE 1:**
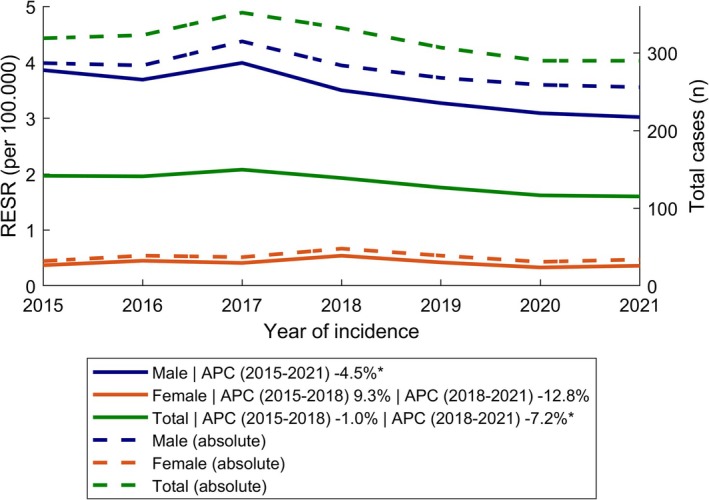
Incidence for early‐stage glottic cancer (2015–2021). Absolute numbers (dashed lines) and RESR (solid lines) in the Netherlands from 2015 to 2021 for the total population (green), males (blue), and females (orange). *Statistically significant. [Color figure can be viewed at wileyonlinelibrary.com]

Figure 1 presents the incidence of early‐stage glottic cancer between 2015 and 2021. The annual number of diagnoses varied, with a peak of 352 cases in 2017 and a minimum of 290 cases in 2020 and 2021. The overall annual incidence demonstrated a decreasing trend, with an average significantly decreasing APC of −7.2% from 2018 to 2021. For male patients, a significant decline was observed over the entire period, with an APC of −4.5%. The APC for female patients did not indicate a statistically significant change.

Between 2015 and 2019, a total of 1633 patients were diagnosed with early‐stage glottic SCC in the Netherlands. The majority were male (88%), and the median age at diagnosis was 69 years (IQR: 62–76 years). Most patients presented with a cT1a tumor (57%), while 18% had cT1b disease and 25% had cT2 disease. At the time of diagnosis, 45% were active smokers. Primary radiotherapy was selected as the initial treatment in 826 cases (51%). In the group receiving radiotherapy, 30% had a cT1a tumor. The median follow‐up for those treated with primary radiotherapy was 67 months (IQR: 52–85 months). Table 1 shows the patient and tumor characteristics.

**TABLE 1:** Patient and tumor characteristics.

Variable	Subgroup	Total		Radiotherapy	
		*N*	%	*N*	%
Total		1633	100	826	100
Gender	Male	1438	88	748	91
	Female	195	12	78	9
Age	< 70	825	53	429	54
	≥ 70	742	47	364	46
Clinical stage	T1a	926	57	249	30
	T1b	292	18	228	28
	T2	415	25	349	42
Year of diagnosis	2015	319	20	157	19
	2016	323	20	171	21
	2017	352	22	188	23
	2018	332	20	167	20
	2019	307	19	143	17
Smoking status	Current smoker	644	45	365	49
	Not smoking at the time of diagnosis	783	55	387	52
Alcohol consumption	Abuse	237	17	138	18
	Social	844	59	427	57
	No consumption at the time of diagnosis	350	24	183	25
Treatment	No treatment	32	2		
	Radiotherapy	826	51		
	Surgery	775	47		
Fractionation schedule	Conventional (2 Gy/fx)^a^			359	47
	Hypofractionated (> 2 Gy/fx)^b^			406	53

^a^ Ninety‐eight percent of the patients received 2 Gy/fx.

^b^ Sixty‐eight percent of the patients received 2.4 Gy/fx, 11% received 2.5 Gy/fx, and 12% received 3.6 Gy/fx.

**FIGURE 2:**
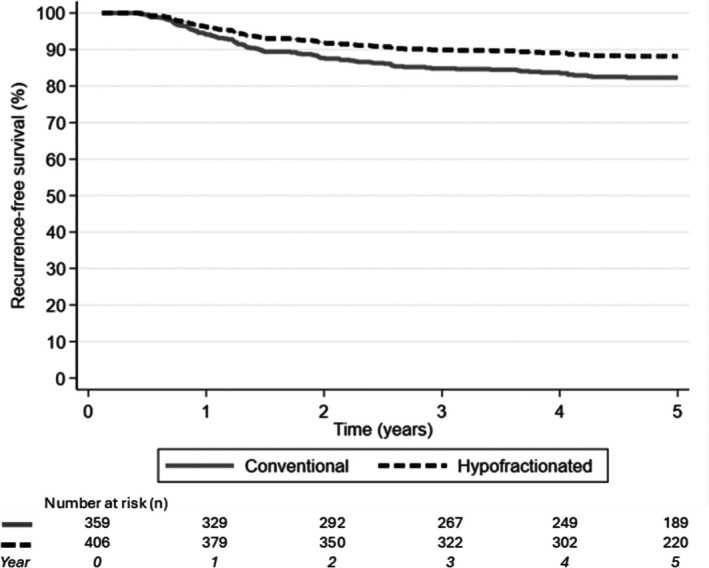
Recurrence‐free survival for conventional and hypofractionated radiation schemes adjusted for age, tumor stage, and smoking status.

After 5 years, 203 patients had died (25%) and 623 patients were alive (75%). The estimated 5‐year overall survival and relative survival were 75% (95% CI: 72%–78%) and 88% (95% CI: 84%–91%), respectively. Overall survival was statistically significantly associated with age, cT‐stage, and smoking status. For relative survival outcomes, a statistically significant association was found for cT‐stage and smoking status (Appendix SA). Ninety patients developed a local recurrence (11%), 22 a regional recurrence (3%), and 5 a distant recurrence (1%) as a first event during 5‐year follow‐up after radiotherapy (Table 2). The 5‐year RFS was 86% (95% CI: 83%–88%). The multivariable Cox regression analysis showed a statistically significant association for fractionation scheme and RFS (HR: 0.6, 95% CI: 0.4–1.0) (Figure 2). Age, smoking, and cT‐stage did not show a statistically significant association with RFS in this multivariable analysis (Table 3). A stratified analysis for tumor stage can be found in Appendix SB.

**TABLE 2:** Number and percentage of patients with a recurrence within 5 years after radiotherapy.

Recurrence	Number of patients	
	*N*	%
Local recurrence	82	9.9
Regional recurrence	14	1.7
Distant recurrence	3	0.4
Local + regional recurrence	7	0.8
Local + distant recurrence	1	0.1
Regional + distant recurrence	1	0.1

**TABLE 3:** Recurrence‐free survival.

Recurrence‐free survival	Univariable			Multivariable		
	HR	95% CI	*p*	HR	95% CI	*p*
Age			**0.03**			0.20
< 70	ref			ref		
≥ 70	0.6	0.4–1.0		0.8	0.5–1.2	
Sex			0.26			
Male	ref	0.3–1.4				
Female	0.6					
cT			**0.003**			0.07
cT1a	ref			ref		
cT1b	1.3	0.7–2.3		1.4	0.8–2.5	
cT2	2.2	1.3–3.5		1.9	1.1–3.2	
Smoking status			**0.02**			0.33
Current smoker	ref			ref		
Not smoking at the time of diagnosis	0.6	0.4–0.9		0.8	0.5–1.2	
Fractionation schedule			**< 0.001**			**0.049**
Conventional	ref			ref		
Hypofractionated	0.5	0.3–0.7		0.6	0.4–1.0	
Alcohol usage			0.89			
Abuse	ref					
Social	0.9	0.6–1.6				
No consumption at the time of diagnosis	0.9	0.5–1.6				

*Note:* The bold values represent the values that are statistically significant.

**FIGURE 3:**
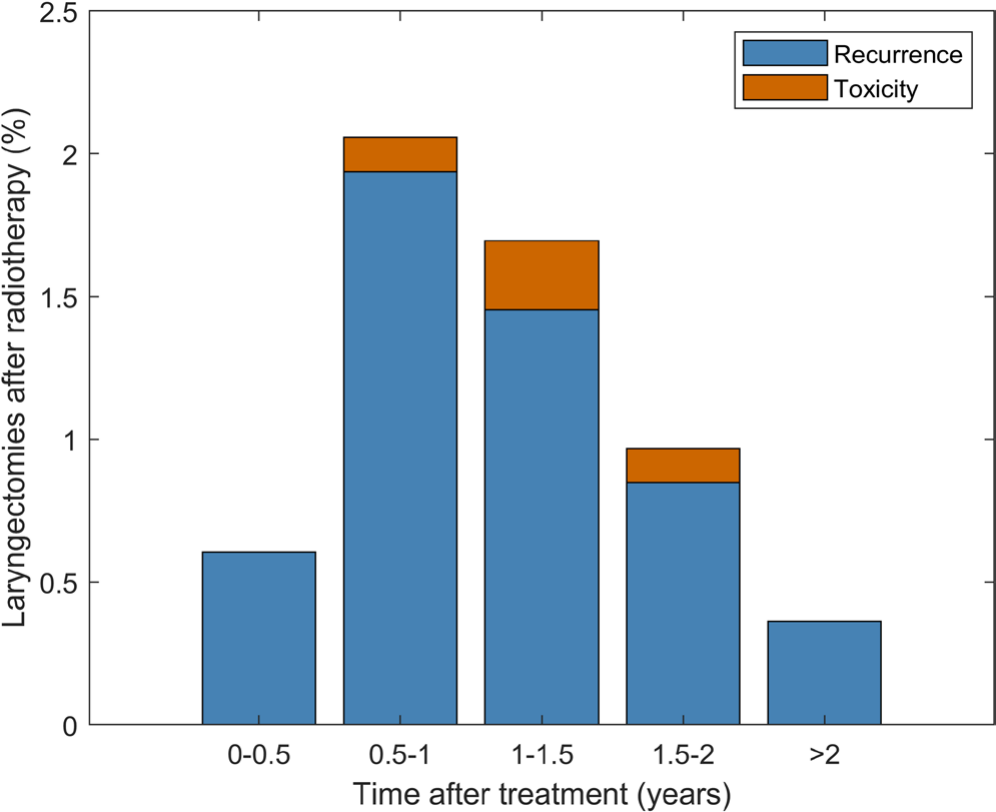
Laryngectomies after radiotherapy treatment. [Color figure can be viewed at wileyonlinelibrary.com]

A total of 47 patients underwent a laryngectomy after radiotherapy treatment (Figure 3). The laryngectomy‐free survival rate was 94% (95% CI: 92%–96%). Among patients who underwent a laryngectomy, a recurrence was found during pathological examination in 43 of 47 patients (91%). The majority of the laryngectomies were conducted within 2 years (44/47 patients; 94%).

Four patients underwent a laryngectomy due to radiation toxicity. The cumulative risk of a laryngectomy due to radiation toxicity at 5 years was 0.5% (95% CI: 0.2–1.4). All four were active smokers at the time of diagnosis and social alcohol consumers. Among them, three had a cT2 tumor, and one had a cT1b tumor. The time between the completion of treatment and the laryngectomy ranged from 7 to 19 months.

## 4 Discussion

This report presents the incidence, survival, recurrences, and laryngectomy rates after radiotherapy treatment for early‐stage glottic cancer in the Netherlands. The incidence of early‐stage glottic cancer decreased between 2015 and 2021. This decline is likely attributed to the stricter smoking policies in the country [12] and is in line with previous research on laryngeal cancer in the Netherlands [13].

Our survival analysis on 826 patients demonstrated an excellent oncological outcome with a 5‐year relative survival of 88%. The relative survival adjusts for mortality in the general population (accounting for age, sex, and year) and therefore approximates cancer‐specific survival. A study using the Surveillance, Epidemiology, and End Results (SEER) database on 3994 early‐stage glottic cancer patients found a cancer‐specific survival of 83.4% [14]. A study using the Danish Head and Neck Cancer Group (DAHANCA) database included 5001 patients diagnosed with glottic SCC between 1971 and 2011 [15]. They reported a 5‐year locoregional failure rate of 15% for cT1 and 32% for T2 stage, while we found that 12.6% of the patients developed a local and/or regional recurrence. This difference may reflect advances in diagnostic and treatment techniques over the years, since our study includes only patients treated between 2015 and 2019. Another, more recent study on the DAHANCA database [16], including only T1a, showed failures in only 6.7% of the patients for an accelerated fractionation scheme. Of these patients, 4.3% received a laryngectomy within 5 years. In our cohort, a laryngectomy was required in 5% of the patients.

For RFS, the hypofractionated group was associated with better outcomes compared to the conventional fractionated group. A single‐center study at UMC Utrecht on patients treated with a 25 × 2.4 Gy fractionation scheme showed a high local control rate (92% at 5 years) [17]. The present study confirmed these positive findings in a nationwide cohort. Higher tumor control probability with hypofractionation is attributed to two factors: larger fraction doses that produce more irreparable DNA damage and a shorter overall treatment time that limits tumor‐cell repopulation between fractions. This mechanism aligns with the improved RFS. Normal tissues also suffer from this increase in irreparable DNA damage. Therefore, the choice to treat patients with a hypofractionated schedule is generally limited to smaller tumors, whereas the conventional schedule is used for the larger cT2 tumors. Thereby, it is suggested to categorize T2 tumors into two groups: without (T2a) and with (T2b) vocal cord impairment [18]. The nationwide Netherlands Cancer Registry does not include information on vocal cord impairment or tumor size. Therefore, these factors could not be included in the present analysis. Tumor stage and smoking status were not found to be associated with the outcomes of the RFS in the multivariable analysis. However, we did find a correlation in the univariable analysis, and multiple single‐center studies did find a correlation between smoking and local control [17, 19].

Laryngectomy after radiotherapy treatment was required in 43 patients (5%) due to recurrence, while only 4 patients (0.5%) underwent laryngectomy as a result of radiation‐induced toxicity. This low incidence of radiation‐induced laryngectomy is consistent with previous studies [20, 21]. In these studies, the radiation‐induced laryngectomy rates were 0.3% and 0.4%. These findings confirm the safety in terms of severe toxicity after radiotherapy treatment in this patient population.

This nationwide cohort provides a comprehensive overview of radiotherapy treatment for early‐stage glottic cancer in the Netherlands. A limitation of the study is the lack of cause‐of‐death data, precluding assessment of disease‐specific survival. Another limitation is that laryngectomy rates were the only available surrogate for severe toxicity. Severe toxicity could also result in other outcomes, such as feeding‐tube or tracheostoma dependence, severe aspiration, or conservatively managed radionecrosis. Current data did not allow for analysis of severe toxicity outcomes other than laryngectomy. Therefore, the incidence of severe toxicity is underestimated.

The excellent oncological outcomes and the low toxicity reflected in the limited occurrence of laryngectomies leave little room for further improvements using conventional treatment strategies. One meaningful opportunity, however, might be the implementation of extreme hypofractionation, which could significantly reduce overall treatment time and reduce the burden on patients and healthcare facilities [22, 23, 24]. The present study sets the benchmark for oncological outcomes and toxicity for future studies.

## 5 Conclusion

This nationwide study demonstrates that radiotherapy for early‐stage glottic SCC results in excellent oncological outcomes. Radiation‐induced laryngectomies for this treatment are extremely rare.

## Author Contributions

**Sabine M. L. Linden:** conceptualization, data curation, formal analysis, investigation, project administration, visualization, writing – original draft. **Boukje A. C. van Dijk:** formal analysis, investigation, methodology, supervision, writing – review and editing. **Marielle E. P. Philippens:** conceptualization, supervision, methodology, writing – review and editing. **Gerben E. Breimer:** conceptualization, supervision, writing – review and editing. **Remco de Bree:** conceptualization, supervision, writing – review and editing. **Johannes A. Rijken:** conceptualization, supervision, writing – review and editing. **Mischa de Ridder:** conceptualization, supervision, methodology, writing – review and editing.

## Funding

This work was supported by Universitair Medisch Centrum Utrecht.

## Conflicts of Interest

The authors declare no conflicts of interest.

## Supporting information


**Appendix SA:** Univariable and multivariable Cox proportional hazard analysis for overall survival and relative survival.

**Appendix SB:** Stratified analysis cT‐stage for recurrence‐free survival.

## Data Availability

Research data are not shared.
