# Supplementary material for: Early‐Stage Glottic Squamous Cell Carcinoma: A Nationwide Analysis on Incidence, Survival, Recurrences, and Laryngectomies After Radiotherapy in the Netherlands (2015–2021)
Source: Head Neck. 2026 Mar 25;48(8):2217–23. doi: 10.1002/hed.70245 (PMC13332543; doi:10.1002/hed.70245)
Supplement: Supplementary file 1 — Appendix SA: Univariable and multivariable Cox proportional hazard analysis for overall survival and relative survival. [file HED-48-2217-s001.pdf]

**Overall survival**		Univariable			Multivariable		
		**HR**	**95% CI**	***p*-value**	**HR**	**95% CI**	***p*-value**
Age	<70	Ref		**<0.001**	ref		**<0.001**
	≥70	2.5	1.9-3.4		3.3	2.4-4.6	
Sex	Male	ref		0.52			
	Female	0.8	0.5-1.4				
cT	cT1a	ref		**<0.001**	ref		**<0.001**
	cT1b	1.2	0.8-1.8		1.1	0.7-1.7	
	cT2	1.9	1.3-2.7		2.0	1.4-2.9	
Smoking status	Current smoker	ref		**0.07**	ref		**<0.001**
	Not smoking at the time of diagnosis	0.8	0.6-1.0		0.6	0.4-0.8	
Fractionation schedule	Conventional	ref		0.75			
	Hypofractionated	1.0	0.7-1.3				
Alcohol consumption	Abuse	ref		0.15			
	Social	0.7	0.5-1.1				
	No consumption at the time of diagnosis	1.0	0.6-1.5				

**Relative survival**		Univariable			Multivariable		
		**RER**	**95% CI**	**p-Value**	**RER**	**95% CI**	**p-Value**
Age	<70	ref		0.95			
	≥70	1.0	0.5-2.0				
Sex	Male	ref		0.77			
	Female	0.8	0.3-2.7				
cT	cT1a	ref		**0.007**	ref		**0.02**
	cT1b	1.1	0.2-7.7		2.0	0.7-5.8	
	cT2	5.3	1.3-21.1		3.3	1.3-8.5	
Smoking status	Current smoker	ref		**0.02**	ref		**0.01**
	Not smoking at the time of diagnosis	0.1	0.0-0.7		0.3	0.1-0.7	
Fractionation schedule	Conventional	ref		0.25			
	Hypofractionated	0.7	0.4-1.3				
Alcohol consumption	Abuse	ref		**0.02**	ref		0.19
	Social	0.3	0.1-0.7		0.6	0.3-1.0	
	No consumption at the time of diagnosis	0.6	0.3-1.3		0.8	0.4-1.5	
