# Supplementary material for: Early‐Stage Glottic Squamous Cell Carcinoma: A Nationwide Analysis on Incidence, Survival, Recurrences, and Laryngectomies After Radiotherapy in the Netherlands (2015–2021)
Source: Head Neck. 2026 Mar 25;48(8):2217–23. doi: 10.1002/hed.70245 (PMC13332543; doi:10.1002/hed.70245)
Supplement: Supplementary file 2 — Appendix SB: Stratified analysis cT‐stage for recurrence‐free survival. [file HED-48-2217-s002.pdf]

cT-stage	Conventional fractionation schedule (n,%)	Hypofractionated fractionation schedule (n,%)	Hazard-ratio fractionation schedule	95% confidence interval
cT1a	68 (29 %)	169 (71%)	0.57	[0.2 1.4]
cT1b	76 (35%)	141 (65%)	0.91	[0.4 1.9]
cT2	215 (69%)	96 (31%)	0.58	[0.3 1.1]
